# Behavioral Modification as a Putative Mediator of Digital Therapeutic Response in Temporomandibular Disorders: Secondary Analysis of a Multicenter Sham-Controlled Randomized Trial

**DOI:** 10.2196/104264

**Published:** 2026-09-03

**Authors:** Soo-Hwan Byun, Sung-Woon On, Byong-Eun Yang, Sung-Ah Che, Sang-Min Yi, Yongjin Park, Yeolib Kim, Sang-Yoon Park

**Affiliations:** 1 Institute of Clinical Dentistry, Hallym University, Hallym University, Chuncheon, Gangwon-do, Republic of Korea; 2 Dental Artificial Intelligence and Robotics R&D Center, Hallym University Medical Center, Anyang, Gyeonggi-do, Republic of Korea; 3 Department of Oral and Maxillofacial Surgery, Hallym University Sacred Heart Hospital, 22, Gwanpyeong-ro 170-gil, Dongan-gu, Anyang, Gyeonggi-do, 14068, Republic of Korea, 82 313805978; 4 Department of Oral and Maxillofacial Surgery, Hallym University Dongtan Sacred Heart Hospital, Hwaseong, Gyeonggi-do, Republic of Korea; 5 Department of Information Systems, College of Business, City University of Hong Kong, Hong Kong, China (Hong Kong); 6 School of Business Administration, Ulsan National Institute of Science and Technology, Ulsan, Ulsan, Republic of Korea

**Keywords:** temporomandibular joint disorders, behavior therapy, digital therapeutics, mediation analysis, randomized controlled trial

## Abstract

**Background:**

Temporomandibular disorders (TMDs) are common chronic conditions involving orofacial pain and functional limitations. Digital therapeutics (DTx) have demonstrated efficacy in TMD management; yet, the behavioral and clinical mechanisms underlying treatment response remain poorly characterized, particularly whether behavioral modification or DTx engagement intensity drives therapeutic benefit.

**Objective:**

This study aimed to investigate the behavioral mechanisms, responder profiles, and moderators of clinical response to a DTx intervention for TMD through a post hoc analysis integrating self-reported, server-derived, and clinician-rated outcome measures.

**Methods:**

We performed a post hoc secondary analysis of a multicenter, double-blind, sham-controlled randomized superiority trial conducted at 2 tertiary care centers in South Korea. The per-protocol cohort comprised 93 participants (DTx: n=44; sham: n=49). Five complementary analyses were applied: responder logistic regression at ≥30%, ≥50%, and ≥70% Visual Analog Scale (VAS) pain-reduction thresholds; subgroup comparison by Oral Behaviors Checklist (OBC) modifier status; causal mediation analysis using the potential outcomes framework with bootstrap CIs; week-4 sensitivity analysis; and moderator analysis testing the treatment×Patient Health Questionnaire-4 (PHQ-4) interaction on VAS change.

**Results:**

DTx assignment was consistently associated with clinically meaningful pain reduction across all 3 responder thresholds (adjusted odds ratios [ORs] 5.39, 95% CI 1.72‐16.94 at ≥30%; 3.21, 95% CI 1.14‐8.99 at ≥50%; and 3.45, 95% CI 1.12‐10.63 at ≥70%; all *P*<.05). Mediation analysis suggested that approximately 29.1% of the total VAS treatment effect may be transmitted via OBC-defined behavioral modification (natural indirect effect −6.91 mm; 95% CI −13.23 to −0.58; *P*=.03), with the mediated proportion rising from 19.6% to 30.2% as responder thresholds became more stringent. Participants with OBC modifiers achieved substantially greater pain reduction than nonmodifiers (−45.71 vs −22.61 mm; difference −23.11; 95% CI −36.97 to −9.24; *P*<.01) despite no significant differences in any objective DTx engagement metric. Treatment ORs were 33%‐42% higher at week 4 than at the 6-week end point (week-4 ORs 7.15, 95% CI 2.39‐21.32 at ≥30%; 4.48, 95% CI 1.68‐11.94 at ≥50%; and 4.91, 95% CI 1.74‐13.87 at ≥70%), suggesting that week 4 may be a candidate time point for future adaptive protocols. Baseline psychological distress (PHQ-4 ≥3) appeared to moderate the treatment response (interaction β=−19.63; 95% CI −37.86 to −1.39; *P*=.04).

**Conclusions:**

Sham-controlled randomized trials in TMD that empirically differentiate behavioral realization from digital engagement volume remain scarce. Behavioral modification, rather than engagement volume, appears to be an important pathway associated with DTx efficacy and may mediate approximately 29% of the pain-reduction effect under exploratory causal assumptions. This advances mechanism-based evaluation of DTx beyond engagement-based surrogates. Week 4 may represent a promising candidate for future adaptive protocols, and baseline psychological distress may warrant investigation for precision patient selection. These preliminary findings should be corroborated by future prospective studies and further mechanistic investigations.

## Introduction

Temporomandibular disorders (TMDs) are common, heterogeneous conditions involving the temporomandibular joint, masticatory muscles, and related structures, typically presenting with orofacial pain and functional limitation [1]. Current models emphasize a biopsychosocial framework where symptom persistence reflects interactions among peripheral factors, central pain modulation, psychological distress, and maladaptive oral behaviors [2-4]. In the diagnostic criteria (DC) for TMDs, Axis II instruments operationalize key psychosocial and behavioral domains; parafunctional habits are clinically relevant because they sustain mechanical loading and pain-guarding cycles [2,5]. Although conservative, reversible, and often multidisciplinary approaches in Axis II are recommended as first-line care [6,7], their effectiveness is often limited by variable adherence to daily behavioral changes outside the clinic [8,9].

Digital therapeutics (DTx) are software-driven, evidence-based therapeutic interventions that have been proposed as a scalable means to deliver standardized behavioral and self-management support and reinforce adherence between clinic visits [10,11]. Over the past decade, DTx have expanded rapidly across medical specialties; an analysis of registered trials documented a more than 5-fold increase since 2011, driven predominantly by mental health and neurological applications [12]. Despite this growth, their development remains unevenly distributed across disciplines and is constrained by a lack of standardized evaluation frameworks, and applications in dentistry and orofacial pain remain scarce [13]. Given the chronic, behaviorally mediated nature of TMD, it is a clinically suitable target for DTx; yet, rigorous evidence in this field has emerged only recently [14,15]. Our group previously reported a multicenter, double-blind, randomized, sham-controlled trial evaluating a TMD DTx intervention, demonstrating clinically meaningful improvements in pain, jaw function, and oral behavior over a 6-week treatment phase [14]. The primary analysis appropriately focused on average between-group effects [14].

However, establishing DTx efficacy is the first step. For clinical implementation and product optimization, it is equally important to understand how benefits are distributed within the treated group and through which modifiable domains contribute to improvement. Digital interventions introduce a persistent black box problem; clinical benefit is plausibly contingent on engagement, but actual exposure varies widely and is not captured by treatment assignment alone [16,17]. Unlike pharmacologic dose, digital dose” is behaviorally expressed and often declines over time, raising questions regarding response heterogeneity and effective behavioral pathways [18]. Schmidt et al [19] have recently proposed a conceptual framework distinguishing “consumed dose” (patient-DTx interaction) from “realized dose” (actual cognitive-affective and behavioral change), arguing that clinical outcomes are driven by dose realization rather than dose intake alone. In this context, an exploratory review of usage data from our randomized controlled trial suggested that greater app use alone was not consistently associated with greater clinical improvement, motivating a more granular, process-level analysis [17]. A better understanding of which behavioral changes accompany the treatment effect may help distinguish therapeutic benefit from engagement volume alone and could inform how these interventions are optimized and applied. Such mechanistic insight may support the progression of TMD DTx from demonstrated efficacy toward more precision-oriented use [19,20].

Accordingly, we conducted a post hoc analysis linking objective DTx use data with repeated clinical and Axis II measures. Such mechanistic analyses remain scarce in TMD DTx research, yet they are essential for moving beyond efficacy demonstrations toward a precision-oriented intervention design. We aimed to (1) identify the clinical and behavioral predictors of response across progressively stringent pain-reduction thresholds, (2) evaluate whether Oral Behaviors Checklist (OBC)–defined behavioral changes mediated the treatment effect, and (3) examine the temporal response dynamics and moderating role of baseline psychological distress. As behavioral change and Axis II domains were not randomized exposures, all analyses were interpreted as associative and hypothesis-generating.

## Methods

### Trial Design

This study is a post hoc analysis of a previously published multicenter, double-blind, randomized, sham-controlled superiority trial (KCT0009493), in which an active TMD DTx app was compared with a visually identical sham app over 6 weeks in a 1:1 allocation at 2 tertiary care oral and maxillofacial surgery clinics in South Korea (Hallym University Sacred Heart Hospital and Hallym University Dongtan Sacred Heart Hospital), with assessments at baseline and weeks 2, 4, and 6 [14]. Detailed eligibility criteria, randomization procedures, intervention content, blinding, ethical approvals, and primary efficacy analyses are reported in the original publication [14]. The present analysis focused on response heterogeneity within the trial by examining predictors of clinically meaningful response, behavioral mediators, and moderators of treatment effect. Patients and members of the public were not involved in the design, conduct, or reporting of the parent trial or the present secondary analysis.

### Sample Size, Randomization, and Blinding

No separate sample size calculation was performed for this post hoc analysis; the analytic sample was determined by the parent trial, which enrolled 102 participants based on a target of 38 participants per group (2-tailed α=.05, 90% power) with an allowance for 25% dropout. In the parent trial, participants were randomly assigned (1:1) by site using stratified block randomization with an independently generated allocation list, and both participants and investigators were blinded to group assignment. The sham application was visually and functionally identical to the active DTx but lacking therapeutic content. Full procedures are reported in the parent trial [14].

### Interventions

Both groups received treatment as usual and were provided with a smartphone app for 6 weeks. The active DTx (Clickless DTx TMD-01) delivered TMD-related education, guided jaw exercises, behavioral habit tracking, guided meditation, and medication guidance with reminder alarms. The sham application was visually and functionally identical in interface and navigation but lacked all therapeutic content, permitting only weekly input of passive user data. Components common to both groups included a jaw health diary for symptom self-monitoring, visual feedback via weekly reports, and supportive check-in calls during weeks 1, 3, and 5, so that the 2 applications differed only in therapeutic content while self-monitoring, feedback, and human contact were held constant. Both applications were locked before trial initiation, and participants accessed them on personal smartphones, with daily use recommended throughout the 6-week period. Full details of intervention development and content are reported in the parent trial and Multimedia Appendix 1 [14].

### Ethical Considerations

This manuscript reports a post hoc secondary analysis of a multicenter, sham-controlled randomized clinical trial. The present secondary analysis was reported in accordance with the CONSORT (Consolidated Standards of Reporting Trials) 2025 guidelines and the CONSORT-EHEALTH (V1.6.1; CONSORT of Electronic and Mobile Health Applications and Online Telehealth, Checklist 1) extension for web-based and mobile health interventions [21,22]. The parent trial was approved by the Institutional Review Boards of Hallym University Sacred Heart Hospital (HALLYM IRB 2024-04-001-006) and Hallym University Dongtan Sacred Heart Hospital (HDT IRB 2024-04-001-004) and was conducted under the supervision of the Korean Ministry of Food and Drug Safety (MFDS) in accordance with the Declaration of Helsinki and the International Council for Harmonization Good Clinical Practice (ICH-GCP) guidelines. The parent trial was prospectively registered on the Clinical Research Information Service (KCT0009493; registration date, May 31, 2024), before enrollment of the first participant on June 20, 2024 [14]. All participants provided written informed consent face-to-face prior to enrollment in the parent trial. The institutional review boards granted an exemption from review for the present secondary analysis of deidentified data, which did not require additional informed consent. Participant data were pseudonymized and stored in a secure, password-protected system accessible only to authorized investigators, with no personally identifiable information included in the analysis. Participants received compensation of approximately KRW 500,000 (Korean won; approximately US $350; KRW 1=US $0.00072 as of June 20, 2024) upon trial completion. All data were managed and locked by an independent contract research organization before the analysis to ensure data integrity and analytical independence. No images in this manuscript or its supplementary materials allow for the identification of individual participants.

### Data Sources

This analysis integrated trial-derived datasets comprising DTx usage logs, module-completion records, and clinical outcome files. Data were cleaned, standardized by participant identifiers and visit labels, and merged using pseudonymized patient IDs. Engagement variables were derived from server logs over the 6-week intervention period and linked to repeated clinical measurements at each scheduled visit.

### Outcomes

App engagement was quantified using objective usage and compliance logs. The variables included total app logins, exercise completion counts, meditation module use, jaw health diary entries, behavioral therapy module interactions, educational video views, article reads, notification checks, and pain record entries, all aggregated over the 42-day intervention period.

Clinical outcomes were assessed at 4 scheduled time points: baseline (V1), week 2 (V2), week 4 (V3), and week 6 (V4; end point). Pain intensity was measured using a Visual Analog Scale (VAS; 0‐100 mm). The jaw function was assessed by a blinded examiner using the maximum mouth opening (MMO; mm) and the Jaw Functional Limitation Scale-20 (JFLS-20). Behavioral and psychological domains were assessed using the OBC and Patient Health Questionnaire-4 (PHQ-4) and scored according to standard procedures for the DC/TMD [23,24].

### Statistical Analysis

Statistical analyses were performed on the per-protocol population (N=93; DTx: n=44; sham: n=49). Because the per-protocol population was defined a priori to exclude participants with missing primary outcome data and no item-level missing data were observed among completers, all analyses were conducted on complete cases without imputation, consistent with the parent trial protocol [14]. Normality was evaluated using the Shapiro-Wilk test. Between-group comparisons were performed using independent-samples *t* tests or Wilcoxon rank-sum tests as appropriate. A 2-sided significance threshold of α=.05 was used. No adjustments for multiple comparisons were made, which was consistent with the exploratory framework, and effect sizes with 95% CIs were reported. Analyses were conducted using Stata (version 17; StataCorp LLC) and Python (version 3.14; Python Software Foundation) with the *pandas, SciPy,* and *Statsmodels* packages.

### Responder Definition and Logistic Regression

Responder status was defined by ≥30%, ≥50%, and ≥70% reduction in pain VAS score from baseline to week 6. Logistic regression models were used to estimate the odds of response for each threshold, with treatment assignment (DTx=1 and sham=0), age, and change in MMO, OBC, and PHQ-4 scores as independent variables. The results were reported as odds ratios (ORs) with 95% CIs.

### OBC Behavioral Modifier Classification

Within the DTx arm (n=44), participants were classified as OBC modifiers (n=21) or nonmodifiers (n=23) based on the median OBC change score from baseline to week 6 (median Δ=−8). Participants with OBC changes below the median (ie, greater behavioral improvement) were designated as modifiers. Independent-samples *t* tests were used to compare clinical outcomes and DTx usage metrics between the subgroups.

### Mediation Analysis

Causal mediation analysis used the potential outcomes framework as implemented in the Mediate command in Stata (StataCorp LLC) [25,26]. The treatment variable was DTx assignment (vs sham), the mediator was OBC modifier status (binary), and the outcomes were (1) continuous VAS change from baseline to week 6 and (2) binary responder status at each threshold. The natural indirect effect (NIE), natural direct effect (NDE), and total effect were estimated using bootstrap-based 95% CIs (1000 iterations). This framework relies on several identifying assumptions, including no unmeasured treatment-mediator, mediator-outcome, or treatment-outcome confounding, no mediator-outcome confounder affected by treatment, and correct model specification. Because treatment was randomized, confounding of the treatment-mediator and treatment-outcome paths was minimized by design, leaving mediator-outcome confounding as the principal residual concern. Given the nonrandomized nature of the mediator, results were interpreted as exploratory and associative rather than confirmatory causal inference.

### Sensitivity and Supplementary Analyses

**Figure 1.**
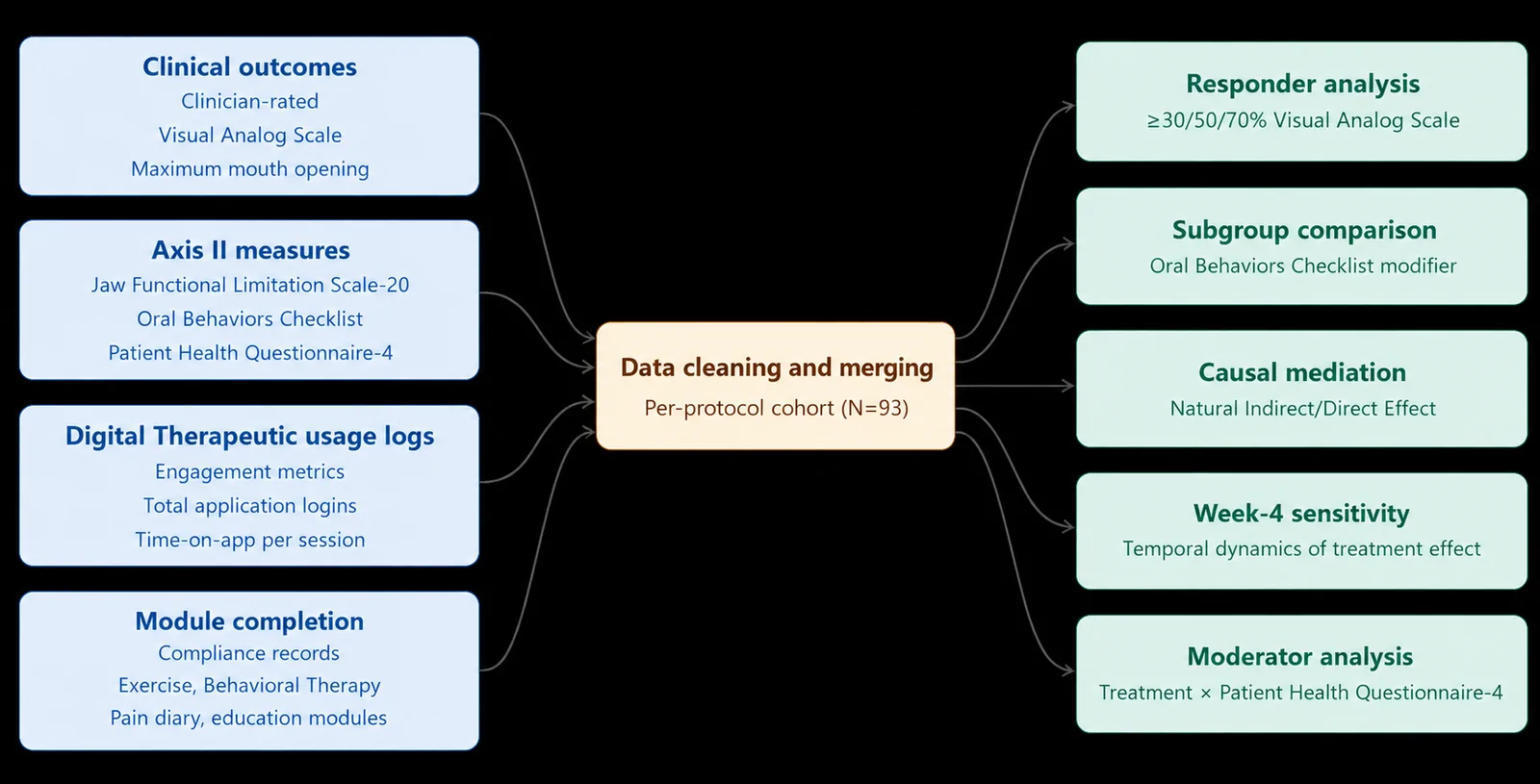
Analytical workflow of the post hoc study. The workflow shows the integration of clinical outcomes, Axis II measures, DTx usage logs, and module-completion records, followed by 5 complementary analyses: responder analysis, OBC modifier subgroup comparison, causal mediation analysis, week-4 sensitivity analysis, and moderator analysis. DC/TMD: Diagnostic Criteria for Temporomandibular Disorders; DTx: digital therapeutic; JFLS-20: Jaw Functional Limitation Scale-20; MMO: maximum mouth opening; OBC: Oral Behaviors Checklist; PHQ-4: Patient Health Questionnaire-4; TMD: temporomandibular disorders; VAS: Visual Analog Scale.

Logistic regression models were reestimated using week 4 change scores to characterize early treatment effects and their temporal dynamics. Probit regression models were used for robustness checks. A linear regression moderator analysis examined the treatment×PHQ-4 High (≥3) interaction on VAS change. The multidomain responder analysis (triple responder: VAS +OBC improvement+MMO improvement) is shown in Multimedia Appendix 2. Figure 1 summarizes the analytical workflow, linking trial-derived data sources to the per-protocol cohort (N=93) and the 5 complementary analyses described below.

## Results

### Study Population and Primary Trial Outcomes

Of the 102 randomized participants, 93 met the per-protocol criteria (DTx: n=44; sham: n=49) and were included in the analysis. As previously reported, the DTx group demonstrated significantly greater improvements at week 6 in VAS pain (between-group difference, −23.78 mm; *P*<.001), MMO (+4.93 mm; *P*<.001), JFLS-20 (−19.68; *P*=.001), and OBC (−5.84; *P*=.008), with no significant difference in PHQ-4 (−0.36; *P*=.40). Baseline characteristics and week-6 outcomes are provided in Multimedia Appendices 3 and 4. Harms were systematically monitored in the parent trial, in which no serious harms or adverse events were reported in either group. The present secondary analysis did not include an additional assessment of harms.

### Factors Associated With Clinically Meaningful Pain Reduction

Across all 3 response thresholds, DTx assignment was a strong predictor of responder status (Table 1). The adjusted OR for DTx was 5.39 (95% CI 1.72‐16.94; *P*<.01) at the ≥30% threshold, 3.21 (95% CI 1.14‐8.99; *P*=.03) at ≥50%, and 3.45 (95% CI 1.12‐10.63; *P*=.03) at ≥70%. MMO improvement was independently associated with higher odds of response at all thresholds (ORs 1.13‐1.15; all *P*<.05). Changes in PHQ-4 scores and age were not significant predictors at any threshold.

**Table 1.** Logistic regression: factors associated with responder status at ≥30%, ≥50%, and ≥70% VAS^a^ reduction thresholds (N=93).

Variable^b^^,c^	≥30%, OR^d^ (95% CI)	*P* value	≥50%, OR (95% CI)	*P* value	≥70%, OR (95% CI)	*P* value
Treatment (DTx^e^=1)	5.39 (1.72-16.94)	<.01^f^	3.21 (1.14-8.99)	.03^f^	3.45 (1.12-10.63)	.03^f^
Age	1.01 (0.97-1.06)	.66	0.99 (0.95-1.03)	.60	0.99 (0.95-1.03)	.64
MMO^g^ change	1.14 (1.03-1.26)	.01^f^	1.15 (1.04-1.27)	<.01^f^	1.13 (1.02-1.26)	.02^f^
OBC^h^ change	0.94 (0.88-0.99)	.03^f^	0.94 (0.89-0.99)	.03^f^	0.91 (0.85-0.98)	.01^f^
PHQ-4^i^ change	1.02 (0.77-1.36)	.87	1.04 (0.79-1.37)	.78	0.77 (0.57-1.03)	.08
Pseudo *R*²	0.27	—^j^	0.25	—	0.32	—

a VAS: Visual Analog Scale.

b Post hoc secondary analysis of a multicenter, double-blind, sham-controlled randomized clinical trial (KCT0009493) evaluating a DTx for temporomandibular disorders, conducted at 2 tertiary care centers in the Republic of Korea from June 2024 to June 2025, with a per-protocol cohort of 93 adults (DTx, n=44; sham, n=49).

c Adjusted odds ratios (95% CI) are shown for treatment assignment, age, and week-6 change in MMO, OBC, and PHQ-4 as factors associated with responder status at ≥30%, ≥50%, and ≥70% VAS pain-reduction thresholds.

d OR: odds ratio.

e DTx: digital therapeutic.

f *P*<.05.

g MMO: maximum mouth opening.

h OBC: Oral Behaviors Checklist.

i PHQ-4: Patient Health Questionnaire-4.

j Not applicable.

OBC change exhibited a threshold-dependent gradient: each unit reduction in OBC score was associated with increased odds of response (OR 0.94 at ≥30% and ≥50%; *P*=.03; OR 0.91 at ≥70%; 95% CI 0.85‐0.98; *P*=.01). The association strengthened as the response criterion became more stringent, with improved model fit (Pseudo *R*²=0.32 at ≥70% vs 0.25-0.27 at lower thresholds). Estimates were robust to model specification, with probit regression yielding directionally consistent treatment effects across all thresholds (Multimedia Appendix 5).

Under a stricter multidomain responder definition requiring simultaneous VAS, OBC, and MMO improvement, DTx participants showed 8.55-12.32-fold higher odds of triple response than sham across thresholds (all *P*<.01; Multimedia Appendix 2).

### OBC Behavioral Modifier Subgroup: Clinical Outcomes and DTx Usage

Within the DTx arm, OBC modifiers (n=21) showed greater pain reduction than nonmodifiers (n=23; VAS change: mean −45.71, SD 17.34 vs mean −22.61, SD 26.75 mm; difference: −23.11 mm; 95% CI −36.97 to −9.24; *P*<0.01) and greater improvement in jaw function (JFLS-20: mean −42.52, SD 26.28 vs mean −22.22, SD 28.72; difference −20.31; 95% CI −37.11 to −3.50; *P*=.02). PHQ-4 improvement did not differ significantly between the subgroups (difference −1.07, 95% CI −2.16 to 0.01; *P*=.05). MMO change also showed no significant difference (difference −0.64, 95% CI −3.88 to 2.61; *P*=.69).

**Figure 2.**
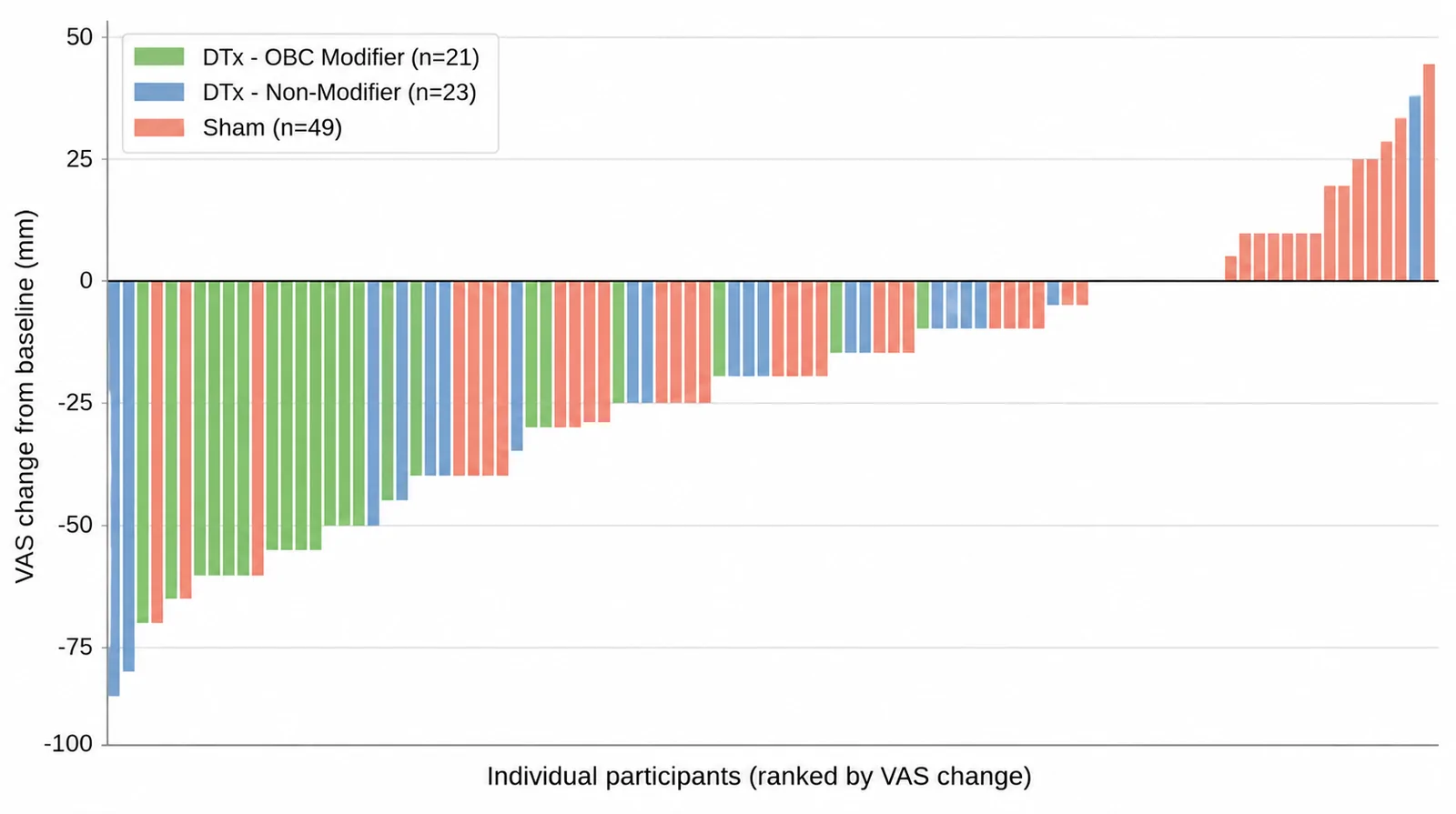
Individual Visual Analog Scale (VAS) changes from baseline ranked by magnitude across treatment subgroups. Each vertical bar represents one participant’s VAS pain change from baseline to week 6, ranked from greatest reduction (left) to smallest reduction or increase (right). Participants are color-coded by treatment allocation and Oral Behaviors Checklist (OBC) modifier status within the digital therapeutic (DTx) arm: DTx-OBC modifier (green; n=21), DTx-nonmodifier (blue; n=23), and sham controls (peach; n=49). Negative values indicate pain reduction. DTx: digital therapeutic; OBC: Oral Behaviors Checklist; TMD: temporomandibular disorders; VAS: Visual Analog Scale.

Despite these clinical differences, no significant between-group differences were observed across any objective DTx usage metric, including total logins, exercise completions, meditation sessions, jaw diary entries, educational video views, or behavioral therapy module uses (all *P*>.05), with a nonsignificant trend toward fewer behavioral therapy entries among the modifiers (*P*=.10; Table 2). The waterfall plot illustrates that the OBC modifier cases were concentrated among participants with the greatest VAS reduction (Figure 2).

**Table 2.** Clinical outcomes and DTx^a^ usage metrics by OBC^b^ modifier status (DTx arm only; n=44).

Variable	OBC modifier^c^ (n=21), mean (SD)	Nonmodifier (n=23), mean (SD)	Difference (95% CI)	*P* value
VAS^d^ change (mm)	–45.71 (17.34)	–22.61 (26.75)	–23.11 (–36.97 to –9.24)	<.01^e^
MMO^f^ change (mm)	6.19 (5.09)	6.83 (5.53)	–0.64 (–3.88 to 2.61)	.69
JFLS-20^g^ change	–42.52 (26.28)	–22.22 (28.72)	–20.31 (–37.11 to –3.50)	.02^e^
PHQ-4^h^ change	–1.33 (1.56)	–0.26 (1.96)	–1.07 (–2.16 to 0.01)	.05
App usage metrics				
Total app logins (42 days)	66.48 (35.31)	61.30 (18.08)	5.17 (–11.67 to 22.02)	.54
Passive exposure				
Educational video	6.86 (5.92)	5.91 (4.98)	0.94 (–2.37 to 4.26)	.57
Article	7.00 (16.02)	7.65 (11.32)	–0.65 (–9.03 to 7.73)	.88
Notification check	4.86 (5.83)	4.48 (5.91)	0.38 (–3.20 to 3.96)	.83
Active practice				
Exercise	133.81 (96.69)	146.43 (86.92)	–12.63 (–68.48 to 43.23)	.65
Meditation	29.71 (46.17)	22.61 (24.85)	7.11 (–15.18 to 29.39)	.52
Self-monitoring and regulation				
Jaw health diary	29.95 (10.95)	32.39 (6.51)	–2.44 (–7.91 to 3.02)	.37
Pain record	4.81 (11.76)	3.83 (7.78)	0.98 (–5.03 to 7.00)	.74
Behavioral therapy	4.29 (7.80)	12.87 (22.20)	–8.58 (–18.91 to 1.74)	.10

a DTx: digital therapeutic.

b OBC: Oral Behaviors Checklist.

c OBC modifiers (n=21) were defined as DTx participants with OBC change scores below the median (ΔOBC=–8, greater behavioral improvement) and were compared with nonmodifiers (n=23) on clinical outcomes and objective engagement metrics using independent-samples *t* tests. For descriptive clarity, engagement metrics are grouped by conceptual domain; all comparisons were performed at the individual metric level.

d VAS: Visual Analog Scale.

e *P*<.05.

f MMO: Maximum Mouth Opening.

g JFLS-20: Jaw Functional Limitation Scale-20.

h PHQ-4: Patient Health Questionnaire-4.

### Mediation Analysis: OBC Behavioral Modification as a Mediator of Treatment Effect

For continuous VAS change, the NIE through OBC modifier status was −6.91 mm (95% CI −13.23 to −0.58; *P*=.03), indicating that approximately 29.1% of the total treatment effect (−23.78 mm) was mediated through behavioral modification. The NDE remained significant (−16.87 mm; 95% CI −27.30 to −6.45; *P*<.01).

**Figure 3.**
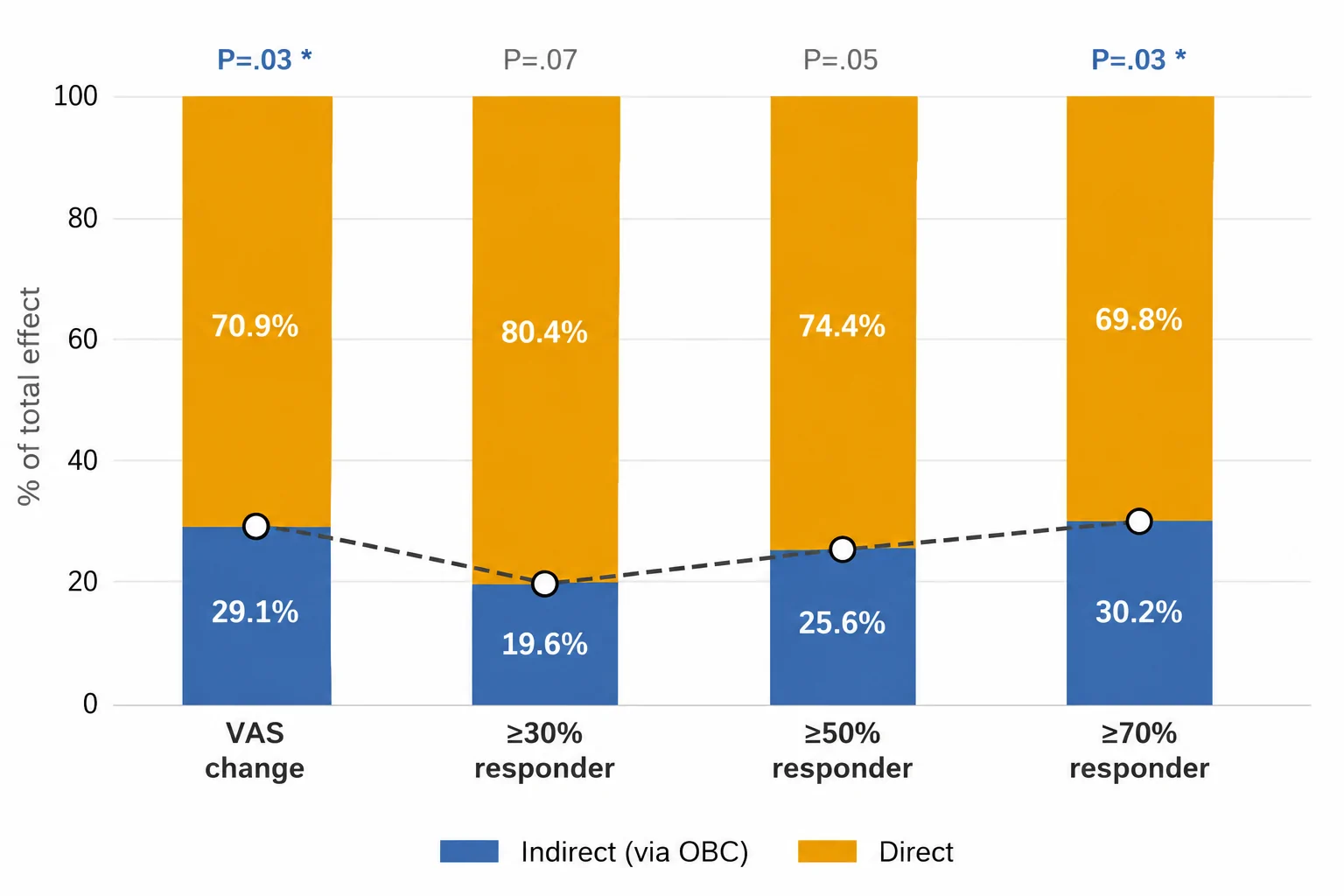
Mediation proportion of Oral Behaviors Checklist (OBC)–driven behavioral change on digital therapeutic (DTx) treatment effects across outcome thresholds. The stacked bars show the proportions of treatment effects mediated through OBC change (indirect) and independent of OBC change (direct) across continuous VAS change and VAS responder thresholds of ≥30%, ≥50%, and ≥70%. DTx: digital therapeutic; OBC: Oral Behaviors Checklist; TMD: temporomandibular disorders; VAS: Visual Analog Scale.

A threshold-dependent pattern emerged for the binary outcomes. The NIE was 0.09 (95% CI −0.01 to 0.18; *P*=.07) at ≥30%, 0.11 (95% CI −0.00 to 0.22; *P*=.05) at ≥50%, and 0.13 (95% CI 0.01‐0.25; *P*=.03) at ≥70%, with the mediated proportion increasing from 19.6% to 25.6% to 30.2%. The NDE remained significant across all models (all *P*<.01; Table 3 and Figure 3).

**Table 3.** Causal mediation analysis: treatment effect on VAS^a^ change and responder status mediated by OBC^b^ behavioral modification (N=93).

Effect^c^	VAS change coefficient (95% CI)	*P* value	≥30% Coeff (95% CI)	*P* value	≥50% Coefficient (95% CI)	*P* value	≥70% Coefficient (95% CI)	*P* value
NIE^d^ (via OBC)	–6.91 (–13.23 to –0.58)	.03^e^	0.09 (–0.01 to 0.18)	.07	0.11 (–0.00 to 0.22)	.05	0.13 (0.01 to 0.25)	.03^e^
NDE^f^ (treatment→outcome)	–16.87 (–27.30 to –6.45)	<.01^e^	0.37 (0.16 to 0.57)	<.01^e^	0.32 (0.11 to 0.53)	<.01^e^	0.30 (0.10 to 0.49)	<.01^e^
Total effect	–23.78 (–33.92 to –13.63)	<.01^e^	0.46 (0.28 to 0.63)	<.01^e^	0.43 (0.24 to 0.61)	<.01^e^	0.43 (0.25 to 0.61)	<.01^e^
Mediation (%)	29.1	—^g^	19.6	—	25.6	—	30.2	—

a VAS: Visual Analog Scale.

b OBC: Oral Behaviors Checklist.

c NIE, NDE, total effect, and proportion mediated are shown for continuous week-6 VAS change and binary responder status at ≥30%, ≥50%, and ≥70% VAS pain-reduction thresholds. Estimates were derived from the potential outcomes framework with 1000-iteration bootstrap 95% CIs.

d NIE: natural indirect effect.

e *P*<.05.

f NDE: natural direct effect.

g Not available.

### Sensitivity Analysis: Early Response at Week 4

**Figure 4.**
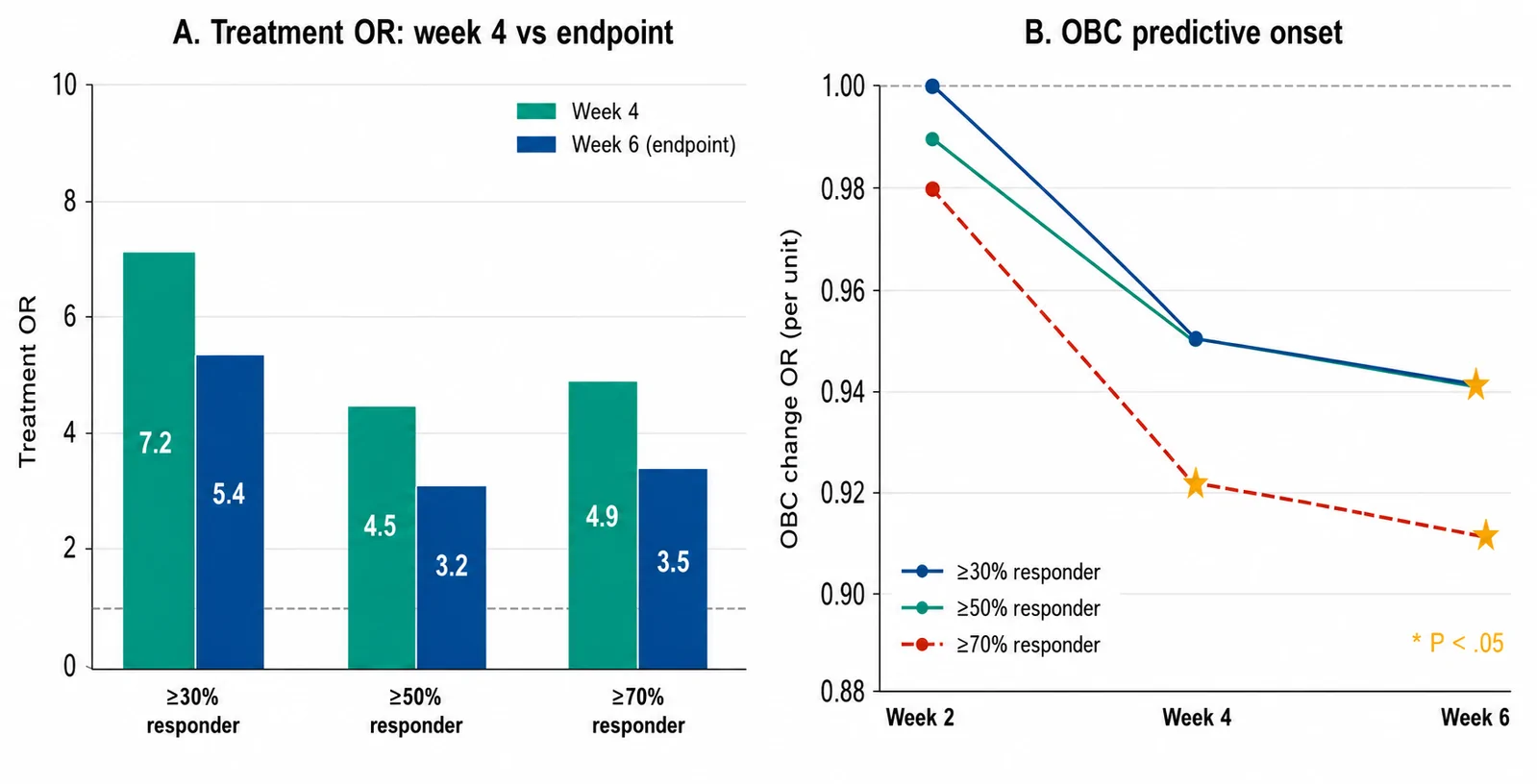
Treatment odds ratios (ORs) at week 4 versus the end point and temporal onset of Oral Behaviors Checklist (OBC) behavioral change across responder thresholds. (A) Treatment odds ratios at week 4 and week 6 across Visual Analog Scale (VAS) pain-reduction responder thresholds of ≥30%, ≥50%, and ≥70%. (B) Temporal changes in the predictive association between OBC behavioral change and VAS responder status across weeks 2, 4, and 6. DTx: digital therapeutic; OBC: Oral Behaviors Checklist; OR: odds ratio; TMD: temporomandibular disorders; VAS: Visual Analog Scale.

When logistic regression models were reestimated using week 4 change scores, treatment ORs were consistently higher than at the 6-week end point across all thresholds: 7.15 (95% CI 2.39‐21.32; *P*<.01) vs 5.39 at ≥30%, 4.48 (95% CI 1.68‐11.94; *P*<.01) vs 3.21 at ≥50%, and 4.91 (95% CI 1.74‐13.87; *P*<.01) vs 3.45 at ≥70%, representing increases of 33%, 40%, and 42%, respectively (Table 4, Figures 4A and 4B).

OBC change at Week 4 was significant for the ≥70% threshold (OR 0.92, 95% CI 0.87‐0.99; *P*=.02) but not at lower thresholds, indicating the behavioral effect matures between weeks 2 and 4. MMO change remained significant across all thresholds at week 4 (ORs 1.10‐1.11; all *P*<.05). The change in PHQ-4 score was not significant in any model. Week-4 estimates were similarly robust under probit regression, with significant treatment effects retained across all thresholds (Multimedia Appendix 6).

**Table 4.** Logistic regression: week 4 (V3-base) change scores as predictors of responder status (N=93).

Variable	≥30%^a^, OR^b^ (95% CI)	*P* value	≥50%, OR (95% CI)	*P* value	≥70%, OR (95% CI)	*P* value
Treatment (DTx^c^=1)	7.15 (2.39-21.32)	<.01^d^	4.48 (1.68-11.94)	<.01^d^	4.91 (1.74-13.87)	<.01^d^
Age	1.01 (0.97-1.05)	.71	0.99 (0.95-1.03)	.60	0.99 (0.95-1.04)	.69
MMO^e^ change (V3-base)	1.10 (1.00-1.21)	.04^d^	1.11 (1.01-1.21)	.03^d^	1.10 (1.00-1.20)	.04^d^
OBC^f^ change (V3-base)	0.95 (0.89-1.01)	.09	0.95 (0.89-1.00)	.07	0.92 (0.87-0.99)	.02^d^
PHQ-4^g^ change (V3-base)	0.99 (0.74-1.33)	.97	0.96 (0.72-1.28)	.79	0.82 (0.61-1.11)	.20
Pseudo *R*²	0.24	—^h^	0.22	—	0.27	—

a Adjusted odds ratios (95% CI) are shown for treatment assignment, age, and week-4 change in MMO, OBC, and PHQ-4 as early treatment predictors of responder status at ≥30%, ≥50%, and ≥70% Visual Analog Scale pain-reduction thresholds. Results are compared with the week-6 end point estimates in Table 1.

b OR: odds ratio.

c DTx: digital therapeutic.

d *P*<.05.

e MMO: Maximum Mouth Opening.

f OBC: Oral Behaviors Checklist.

g PHQ-4: Patient Health Questionnaire-4.

h Not applicable.

### Exploratory Moderator Analysis: Baseline Psychological Distress

To explore whether baseline psychological distress moderated the treatment effect, a linear regression model with an interaction term (Treatment×PHQ-4 High [≥3]) was used with VAS change as the dependent variable (Table 5). A PHQ-4 score of ≥3 was applied as the binary threshold, consistent with established cutoff criteria indicating clinically meaningful anxiety and/or depressive symptoms [27,28]. Of the 93 participants, 65 had PHQ-4 ≥3 and 28 had PHQ-4 <3 at baseline.

**Figure 5.**
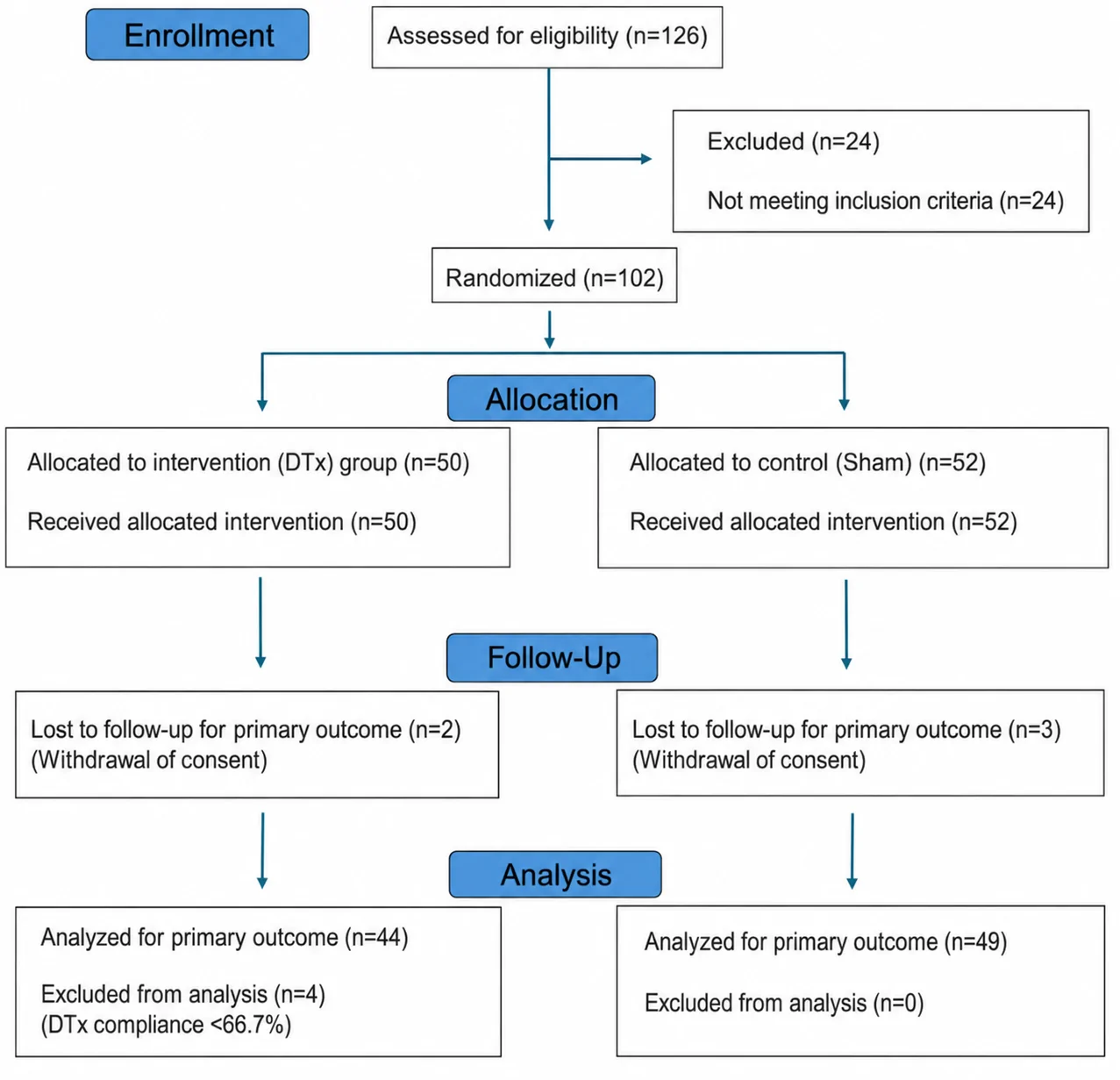
CONSORT (Consolidated Standards of Reporting Trials) flow diagram of the randomized controlled trial. DTx: digital therapeutic.

In the adjusted model controlling for baseline VAS, age, and sex, the interaction term was significant (β=−19.63; *P*=.04), indicating the DTx effect on VAS change differed by baseline distress level. Among participants with PHQ-4 ≥3, the estimated DTx effect on VAS change was −28.09 mm (sum of treatment coefficient and interaction term: −8.46 + [−19.63]), representing greater pain reduction compared with those with PHQ-4 <3, in whom no significant treatment effect was observed (β=−8.46 mm; *P*=.25). The main effect of PHQ-4 High was significant (*β*=14.91; *P*=.01), indicating that higher baseline psychological distress was independently associated with greater VAS reduction regardless of treatment assignment. The adjusted model explained 49% of the variance in VAS score change (*R*²=0.49), compared with 24% in the unadjusted model (*R*²=0.24). As a sensitivity analysis, the interaction was not significant when PHQ-4 was modeled as a continuous variable (β=−4.02 per point; *P*=.06). The CONSORT flow diagram for the overall clinical trial is presented in Figure 5.

**Table 5.** Moderator analysis: treatment×baseline Patient Health Questionnaire-4 (PHQ-4) interaction on Visual Analog Scale (VAS) change (N=93).

Variable^a^	Unadjusted β (SE)	95% CI	*P* value	Adjusted β (SE)	95% CI	*P* value
Treatment ×PHQ-4 High	–26.62 (10.75)	–47.98 to –5.26	<.01^b^	–19.63 (9.17)	–37.86 to −1.39	.04^b^
Treatment (DTx^c^=1)	–7.15 (8.65)	–24.33 to 10.02	.41	–8.46 (7.34)	–23.04 to 6.12	.25
PHQ-4^d^ High (≥3)	16.00 (7.07)	(1.95 to 30.05)	.03^b^	14.91 (5.97)	3.04 to 26.77	.01^b^
VAS^e^ baseline	—^f^	—		–0.68 (0.11)	–0.90 to –0.46	<.01^b^
Age	—	—		0.29 (0.18)	–0.07 to 0.65	.11
Sex (female=1)	—	—		–6.14 (4.66)	–15.32 to 3.03	.19
*R*²	0.24	—		0.49	—	

a Linear regression estimates (β, SE, and 95% CI) are shown for the Treatment×PHQ-4 High (≥3) interaction on VAS change from baseline to week 6, adjusted for baseline VAS, age, and sex. Baseline PHQ-4 ≥3 (n=65) versus PHQ-4 <3 (n=28) was used as a validated cut-point indicating clinically meaningful anxiety and/or depressive symptoms.

b P<.05.

c DTx: digital therapeutic.

d PHQ-4: Patient Health Questionnaire-4.

e VAS: Visual Analog Scale.

f Not available.

## Discussion

### Principal Findings

This post hoc analysis of a multicenter, sham-controlled randomized trial of a TMD DTx extends the parent trial’s demonstration of aggregate efficacy to the mechanisms and heterogeneity of treatment response. Building on the confirmed clinical benefit of the DTx, we examined which clinical and behavioral factors were associated with pain response, whether oral behavior change might mediate the treatment effect, and how the treatment effect evolved over time and varied with baseline psychological distress. Across all response thresholds, DTx assignment remained a strong and consistent predictor of clinically meaningful pain relief relative to sham. Three exploratory patterns emerged, including oral behavior modification appeared as a possible mediating pathway, the treatment effect appeared to be accelerated and amplified, with responders reaching clinically meaningful pain relief earlier and to a greater degree, and baseline psychological distress may play a modest moderating role. As all analyses were performed post hoc, these patterns were interpreted as associative and hypothesis-generating and are elaborated below.

In the present analysis, OBC change was the most consistent independent factor associated with response, with the association strengthening at progressively more stringent response thresholds. This gradient may indicate that behavioral modification becomes increasingly important as pain relief increases. Mediation analysis was consistent with this pattern. The proportion of the VAS treatment effect potentially transmitted via behavioral modification rose monotonically across increasingly stringent response thresholds, reaching approximately 29% on the continuous VAS scale. This positive dose-response relationship, in which greater behavioral change accompanied greater pain reduction, is consistent with OBC behavioral modification potentially acting as a partial mediating pathway rather than solely as a correlate.

A particularly informative pattern was the dissociation between OBC-defined behavioral change and objective app usage metrics. OBC modifiers achieved significantly greater pain reduction than nonmodifiers, yet no significant differences were observed across any engagement metrics, including logins, exercises, meditation sessions, or diary entries. The nonsignificant but directionally consistent pattern of fewer behavioral therapy violation entries among modifiers provides a triangulation across 3 independent measurement modalities, including self-report OBC, digital behavioral logs, and clinical pain VAS outcomes. Of note, the behavioral therapy module log records patient-reported breaches of behavioral recommendations; therefore, fewer entries indicate better behavioral adherence rather than reduced app use. This directionality aligns the digital log with the lower OBC scores and the greater pain reduction observed in modifiers, with all 3 modalities converging on the same pattern. This suggests that meaningful behavioral change, rather than passive app engagement, may be the more relevant driver of clinical improvement in this cohort [19]. This dissociation should nonetheless be interpreted cautiously, as the present sample may be underpowered to detect modest differences in engagement metrics.

Treatment ORs were consistently higher at week 4 than at the 6-week end point across all responder thresholds, which may suggest that DTx had its strongest discriminatory effect at this intermediate time point. The temporal onset of OBC as an associated marker of response, which was absent at week 2 but emerged for the ≥70% threshold by week 4, may suggest that meaningful behavioral modification requires approximately 4 weeks to consolidate and manifest clinically [29]. Taken together, these temporal patterns may suggest that DTx responders achieve an accelerated and amplified clinical improvement than nonresponders, reaching clinically meaningful pain relief earlier and to a greater degree, with behavioral modification as a plausible temporal correlate of this response.

Baseline psychological distress (PHQ-4 ≥3) appeared to modestly amplify the treatment effect, with a larger estimated DTx effect in the elevated-distress subgroup than in the subgroup without distress. However, the interaction was not significant when PHQ-4 was modeled continuously, and the ≥3 threshold was a predefined clinical cutoff rather than a data-derived optimum; these findings are exploratory and require prospective validation [27].

### Behavioral Mechanism and the Realized-Dose Framework

The central role of OBC behavioral change aligns with and extends the biopsychosocial understanding of TMD pathophysiology [30]. Parafunctional oral behaviors, including clenching, bruxism, and habitual jaw tension, are recognized perpetuating factors that sustain mechanical loading on the temporomandibular joint and maintain pain-guarding cycles [30,31]. Longitudinal evidence from the Orofacial Pain: Prospective Evaluation and Risk Assessment (OPPERA) study associated high awake parafunction with both TMD onset and chronification [24,32]. Building on this prevalence-level evidence, our findings suggest that targeted modification of these parafunctional behaviors may yield measurable clinical improvement, moving the inference beyond a statistical between-group comparison toward a data-based mechanistic account of how parafunction-directed intervention may translate into pain reduction.

This dissociation is conceptually consistent with the framework proposed by Schmidt et al [19], who distinguish between “consumed dose,” the patient’s interaction with a DTx, and “realized dose,” the degree to which cognitive-affective and behavioral change is actually internalized by the patient, and who argue that the therapeutic effect is not a direct function of consumed dose but rather of realized dose. This convergence is consistent with the view that therapeutic benefit may depend more on behavioral adaptation, the realized dose, than on engagement volume [19]. It thus raises questions about the assumption that a greater consumed dose should predict clinical outcomes [18]. Our results provide a data-based, exploratory quantification of this distinction within a sham-controlled randomized clinical trial, suggesting that OBC-defined behavioral modification may account for approximately 29% of the total VAS treatment effect independently of any consumed dose metric. For DTx product development, optimization might prioritize facilitating real-world behavioral internalization through adaptive content delivery and habit-change reinforcement rather than maximizing session frequency or time-on-app [33-35]. These findings further caution against the use of engagement metrics such as login frequency or session duration, as surrogate indicators of adherence or therapeutic dose, given that meaningful behavioral change may occur independently of, or even inversely to, raw usage volume [19].

### Temporal Dynamics of Response and Moderation by Psychological Distress

Because week-4 behavioral change precedes the week-6 end point, it can be interpreted as an early-treatment predictor with temporal precedence, in contrast to the concurrent change variables measured at the end point, which are more appropriately regarded as factors associated with response. The week-4 amplification has clear precedent in the broader habit-formation literature, where behavioral consolidation typically requires approximately 4 weeks to stabilize [29]. These findings may carry 2 practical implications. First, week 4 may represent a promising candidate time point at which patients who have not demonstrated a behavioral or clinical response by this time point can be identified early to receive augmented or alternative strategies before completing the full 6-week course. However, because these observations are post hoc, prospective adaptive-design or decision-analytic studies would be needed before a week-4 rule could inform clinical practice [36]. These adaptive treatment approaches are increasingly advocated within digital health interventions [36]. Second, the amplified ORs raise the prospective hypothesis that a condensed 4-week protocol might achieve equivalent or near-equivalent gains with reduced patient burden, a question that warrants dedicated evaluation in future trials [37].

The PHQ-4 moderator pattern is clinically plausible: patients with psychological comorbidity may benefit more from the self-management, mindfulness, and behavioral awareness components in the DTx intervention [38-40]. Despite evidence that 60%‐77% of patients with TMD experience psychological distress [3], routine screening remains underused in dental practice [41]. Brief instruments such as the PHQ-4 could help identify patients most likely to benefit from behavioral digital interventions [42,43].

### Limitations

This study has several limitations. All findings are from a post hoc secondary analysis and are associative and hypothesis-generating, not causal. Because the proposed mediator, OBC-defined behavioral change, is a postrandomization variable rather than a randomized exposure, causal mediation inference is precluded despite the use of the potential outcomes framework, and residual unmeasured confounding of the mediator-outcome relationship cannot be excluded [44]. In particular, reverse causation cannot be ruled out: patients whose pain improves may subsequently report fewer parafunctional behaviors because they feel better, are less vigilant, or perceive less need to guard the jaw [3]. Under this scenario, OBC improvement could function as both a consequence and a correlate of pain reduction rather than solely as an antecedent mediator, and the assumed direction from behavioral change to pain relief, while biologically plausible, cannot be confirmed in the present design. Baseline OBC and PHQ-4 scores were higher in the DTx group, which may have influenced the mediation and moderator analyses through regression to the mean. The OBC modifier classification relies on a data-driven median split, introducing cut-point sensitivity, and creating subgroups that may not be stable across samples. Furthermore, multiple responder, subgroup, mediation, sensitivity, and moderator analyses were conducted without formal correction for multiple comparisons; borderline findings, particularly those with *P* values close to .05, should therefore be regarded as exploratory signals rather than confirmatory results. Finally, the mediation estimates were sensitive to distributional assumptions and sample sizes, necessitating replication in larger independent cohorts [44].

The analysis was conducted in the per-protocol population (N=93) rather than the intention-to-treat sample, as the mechanistic question of how DTx exerts its therapeutic effect inherently concerns participants who completed the intervention as intended; intention-to-treat estimates would have diluted the engagement-outcome dissociation with noncompleters and were therefore not appropriate for this specific analytic aim [45]. This principled selection nonetheless restricts inference to completers and should be considered when generalizing the mechanistic findings to broader trial populations. The per-protocol sample size also restricted the statistical power of the subgroup and interaction analyses, particularly for the PHQ-4 moderator, and limited the ability to detect modest between-group differences in engagement metrics. In addition, only a single behavioral mediator (OBC) was examined; other plausible pathways, such as cognitive reappraisal, pain-related self-efficacy, or improved sleep, were not assessed and may also contribute to treatment response [46-48]. The 6-week follow-up precluded the assessment of long-term durability, and a longitudinal follow-up study is currently underway. The trial was conducted at 2 tertiary care centers in South Korea, limiting its generalizability to other health care settings and populations.

### Conclusion

These findings may refine the understanding of therapeutic dose in digital interventions. While app engagement may be a necessary vehicle, it may be the patient’s internalization of that engagement into sustained behavioral change that is more closely linked to clinical benefit. In TMD, this realized behavioral adaptation, shaped by underlying psychological context and consolidated within a defined temporal window, may represent a key behavioral pathway underlying treatment response. Practically, these observations suggest that DTx development and evaluation might prioritize measures of behavioral realization over engagement volume and that mechanism-driven, precision-oriented trial designs may help clarify for whom and through which pathways these interventions work. These directions remain hypotheses to be confirmed in future prospective studies.

## Supplementary material

10.2196/104264 Multimedia Appendix 1 Structure and components of the active digital therapeutic and sham applications in the parent randomized trial.

10.2196/104264 Multimedia Appendix 2 Multidomain responder analysis: triple responder rates by treatment group triple responder=simultaneous improvement in all 3 domains: VAS pain reduction (≥threshold)+OBC improvement (ΔOBC ≤ median −4)+MMO improvement (ΔMMO≥median+4).

10.2196/104264 Multimedia Appendix 3 Baseline demographic and clinical characteristics (per-protocol population).

10.2196/104264 Multimedia Appendix 4 Change From Baseline to Week 6: DTx vs. Sham Group.

10.2196/104264 Multimedia Appendix 5 Probit regression analysis of VAS responder status at week 6 (end point)-robustness check.

10.2196/104264 Multimedia Appendix 6 Probit regression analysis of VAS responder status at week 4-sensitivity analysis.

10.2196/104264 Checklist 1 Checklist 1: CONSORT-EHEALTH checklist (V1.6.1).

## Abbreviations

CONSORT: Consolidated Standards of Reporting Trials
CONSORT-EHEALTH: Consolidated Standards of Reporting Trials of Electronic and Mobile Health Applications and Online Telehealth
DC/TMD: Diagnostic Criteria for Temporomandibular Disorders
DTx: digital therapeutic
ICH-GCP: International Council for Harmonization Good Clinical Practice
JFLS-20: Jaw Functional Limitation Scale-20
MFDS: Korean Ministry of Food and Drug Safety
MMO: maximum mouth opening
NDE: natural direct effect
NIE: natural indirect effect
OBC: Oral Behaviors Checklist
OPPERA: Orofacial Pain: Prospective Evaluation and Risk Assessment
OR: odds ratio
PHQ-4: Patient Health Questionnaire-4
TMD: temporomandibular disorder
VAS: Visual Analog Scale
