# Supplementary material for: Behavioral Modification as a Putative Mediator of Digital Therapeutic Response in Temporomandibular Disorders: Secondary Analysis of a Multicenter Sham-Controlled Randomized Trial
Source: JMIR Mhealth Uhealth. 2026 Sep 3;14:e104264. doi: 10.2196/104264 (PMC13541226; doi:10.2196/104264)
Supplement: Supplementary file 2 [file mhealth-v14-e104264-s002.docx]

**Multimedia appendix 2.** Multi-domain Responder Analysis: Triple Responder Rates by Treatment Group

Triple responder = simultaneous improvement in all three domains: VAS pain reduction (≥threshold) + OBC improvement (ΔOBC ≤ median −4) + MMO improvement (ΔMMO ≥ median +4)

|  | | **DTx (n=44)** | **Sham (n=49)** | **OR (95% CI)** | **χ²** | ***P* value** |
| --- | --- | --- | --- | --- | --- | --- |
| **Component responders** |  | |  |  |  |  |
| **VAS ≥30% responder** | 38 (86.4%) | | 20 (40.8%) | 9.50 (3.28–27.51) |  | <0.01*** |
| **OBC responder** | 30 (68.2%) | | 20 (40.8%) | 3.11 (1.32–7.35) |  | 0.01*** |
| **MMO responder** | 32 (72.7%) | | 18 (36.7%) | 4.59 (1.89–11.13) |  | <0.01*** |
| **Triple responders** |  | |  |  |  |  |
| **VAS ≥30% + OBC + MMO** | 23 (52.3%) | | 4 (8.2%) | 12.32 (3.79–40.07) | 21.89 | <0.01*** |
| **VAS ≥50% + OBC + MMO** | 21 (47.7%) | | 4 (8.2%) | 10.27 (3.16–33.37) | 18.46 | <0.01*** |
| **VAS ≥70% + OBC + MMO** | 19 (43.2%) | | 4 (8.2%) | 8.55 (2.58–28.34) | 15.27 | <0.01*** |

Data are n (%). OR = odds ratio. Component responder ORs estimated by chi-square; triple responder ORs calculated from 2×2 contingency tables. All p values from Pearson chi-square test. **P<0.05.*
