# Supplementary material for: Behavioral Modification as a Putative Mediator of Digital Therapeutic Response in Temporomandibular Disorders: Secondary Analysis of a Multicenter Sham-Controlled Randomized Trial
Source: JMIR Mhealth Uhealth. 2026 Sep 3;14:e104264. doi: 10.2196/104264 (PMC13541226; doi:10.2196/104264)
Supplement: Supplementary file 3 [file mhealth-v14-e104264-s003.docx]

**Multimedia appendix 3.** *Baseline Demographic and Clinical Characteristics (Per-Protocol Population)*

| **Variable** | **DTx Group (n=44) Mean (SD)** | | **Sham Group (n=49) Mean (SD)** | **Difference (95% CI)** | ***P* value** |
| --- | --- | --- | --- | --- | --- |
| Sex (female) | 0.70 (0.46) | | 0.65 (0.48) | 0.05 (−0.14, 0.25) | 0.62 |
| Age (years) | 36.86 (11.67) | | 40.63 (12.36) | −3.77 (−8.74, 1.20) | 0.13 |
| VAS (mm) | 50.57 (22.13) | | 47.55 (17.41) | 3.02 (−5.14, 11.18) | 0.46 |
| MMO (mm) | 40.55 (6.92) | | 39.55 (7.90) | 0.99 (−2.08, 4.07) | 0.53 |
| JFLS-20 (sum) | 47.91 (32.10) | | 46.12 (23.41) | 1.79 (−9.71, 13.28) | 0.76 |
| OBC (sum) | 26.68 (9.13) | | 22.12 (9.27) | 4.56 (0.76, 8.36) | 0.02* |
| PHQ-4 (sum) | 3.68 (2.30) | 2.78 (2.00) | | 0.91 (0.02, 1.79) | 0.04* |

DTx: digital therapeutic; VAS: Visual Analog Scale; MMO: maximum mouth opening; JFLS-20: Jaw Functional Limitation Scale-20; OBC: Oral Behaviors Checklist; PHQ-4: Patient Health Questionnaire-4. *P<0.05.
