# Supplementary material for: Behavioral Modification as a Putative Mediator of Digital Therapeutic Response in Temporomandibular Disorders: Secondary Analysis of a Multicenter Sham-Controlled Randomized Trial
Source: JMIR Mhealth Uhealth. 2026 Sep 3;14:e104264. doi: 10.2196/104264 (PMC13541226; doi:10.2196/104264)
Supplement: Supplementary file 4 [file mhealth-v14-e104264-s004.docx]

**Multimedia appendix 4.** *Change From Baseline to Week 6: DTx vs. Sham Group*

| **Outcome** | **DTx (n=44)**  **Mean Change (SD)** | **Sham (n=49)**  **Mean Change (SD)** | **Difference (95% CI)** | ***P* value** |
| --- | --- | --- | --- | --- |
| **VAS (mm)** | −33.64 (25.34) | −9.86 (24.96) | −23.78 (−34.15, −13.41) | <0.001*** |
| **MMO (mm)** | +6.52 (5.27) | +1.59 (6.21) | +4.93 (2.54, 7.32) | <0.001*** |
| **JFLS-20 (sum)** | −31.91 (29.13) | −12.22 (27.16) | −19.68 (−31.28, −8.09) | 0.001*** |
| **OBC (sum)** | −7.84 (12.56) | −2.00 (7.47) | −5.84 (−10.05, −1.63) | 0.008*** |
| **PHQ-4 (sum)** | −0.77 (1.84) | −0.41 (2.15) | −0.36 (−1.19, 0.46) | 0.40 |

Positive values for MMO indicate improvement (increased opening). Negative values for VAS, JFLS-20, OBC, and PHQ-4 indicate improvement (score reduction). *P<0.05.
