# Supplementary material for: Behavioral Modification as a Putative Mediator of Digital Therapeutic Response in Temporomandibular Disorders: Secondary Analysis of a Multicenter Sham-Controlled Randomized Trial
Source: JMIR Mhealth Uhealth. 2026 Sep 3;14:e104264. doi: 10.2196/104264 (PMC13541226; doi:10.2196/104264)
Supplement: Supplementary file 5 [file mhealth-v14-e104264-s005.docx]

**Multimedia appendix 5.** Probit Regression Analysis of VAS Responder Status at Week 6 (Endpoint) -Robustness Check

| **Variable** | **≥30% OR (95% CI)** | ***p* value** | **≥50% OR (95% CI)** | ***P* value** | **≥70% OR (95% CI)** | ***P* value** |
| --- | --- | --- | --- | --- | --- | --- |
| Treatment (DTx=1) | 0.78 (0.33) | 0.02*** | 0.98 (0.33) | <0.01*** | 0.71 (0.31) | 0.02*** |
| Age | −0.01 (0.01) | 0.57 | 0.00 (0.01) | 0.75 | −0.01 (0.01) | 0.55 |
| MMO change | 0.07 (0.03) | 0.01*** | 0.08 (0.03) | 0.01*** | 0.08 (0.03) | <0.01*** |
| OBC change | −0.05 (0.02) | <0.01*** | −0.04 (0.02) | 0.02*** | −0.04 (0.02) | 0.02*** |
| PHQ-4 change | −0.17 (0.09) | 0.07 | 0.01 (0.09) | 0.89 | 0.02 (0.09) | 0.82 |
| Constant | −1.12 (0.58) | 0.05 | −0.63 (0.55) | 0.26 | −0.37 (0.54) | 0.49 |
| Pseudo R² | 0.32 |  | 0.28 |  | 0.25 |  |

Coefficients are marginal probit estimates. OBC change and MMO change are from baseline to week 6 (endpoint). PHQ-4, Patient Health Questionnaire-4; OBC, Oral Behaviors Checklist; MMO, maximum mouth opening; SE, standard error. *P<.05.
