# Supplementary material for: Behavioral Modification as a Putative Mediator of Digital Therapeutic Response in Temporomandibular Disorders: Secondary Analysis of a Multicenter Sham-Controlled Randomized Trial
Source: JMIR Mhealth Uhealth. 2026 Sep 3;14:e104264. doi: 10.2196/104264 (PMC13541226; doi:10.2196/104264)
Supplement: Supplementary file 6 [file mhealth-v14-e104264-s006.docx]

**Multimedia appendix 6. Probit Regression Analysis of VAS Responder Status at Week 4 - Sensitivity Analysis**

| **Variable** | **≥30% OR (95% CI)** | ***P* value** | **≥50% OR (95% CI)** | ***P* value** | **≥70% OR (95% CI)** | ***P* value** |
| --- | --- | --- | --- | --- | --- | --- |
| **Treatment (DTx=1)** | 0.96 (0.31) | <0.01*** | 1.16 (0.32) | <0.01*** | 0.91 (0.30) | <0.01*** |
| Age | −0.01 (0.01) | 0.64 | 0.00 (0.01) | 0.85 | −0.01 (0.01) | 0.53 |
| MMO change | 0.06 (0.03) | 0.04 | 0.06 (0.03) | 0.05 | 0.06 (0.03) | 0.02*** |
| OBC change | −0.05 (0.02) | 0.01*** | −0.03 (0.02) | 0.07 | −0.03 (0.02) | 0.06 |
| PHQ-4 change | −0.13 (0.09) | 0.17 | −0.01 (0.09) | 0.92 | −0.03 (0.09) | 0.74 |
| Constant | −1.02 (0.56) | 0.07 | −0.49 (0.54) | 0.36 | −0.30 (0.52) | 0.57 |
| Pseudo R² | 0.28 |  | 0.24 |  | 0.22 |  |

Coefficients are marginal probit estimates. OBC change and MMO change are from baseline to week 4 (V6). PHQ-4, Patient Health Questionnaire-4; OBC, Oral Behaviors Checklist; MMO, maximum mouth opening; SE, standard error. *P<0.05.
